# Quality culture, university-industry collaboration, and perceived employability among vocational students in China: a Yanpei Huang perspective

**DOI:** 10.3389/fpsyg.2024.1439097

**Published:** 2024-08-16

**Authors:** Hui Li, Shoukat Iqbal Khattak, Muhammed Asif Shamim

**Affiliations:** ^1^Research Institute of Vocational Education, Xiamen City University, Xiamen, China; ^2^School of Business Administration, Jimei University, Xiamen, China; ^3^Department of Business Administration, Iqra University, Karachi, Pakistan

**Keywords:** university quality culture, student’s perceived employability, Yanpei Huang, China, university-industry collaboration

## Abstract

Students’ perceived employability (SPE) can be seen as one of the indicators of technical and vocational education and training (TVET) quality. However, less is known about the determining factors of SPE in vocational education. As the founder of modern vocational education in China, Yanpei Huang has written a large volume on ensuring students’ employability and the quality culture of TVET. Nowadays, as the feature and nature of TVET, university-industry collaboration (UIC) has been promoted worldwide. The primary objective of this study was to investigate the influence of quality culture from Yanpei Huang’s perspective (YHQC) on the SPE and the UIC role in the relationship between YHQC and SPE in a TVET university in China. Data were collected by questionnaire from 341 students from one vocational education university in China. The questionnaire included measures of quality culture, perceived employability, and UIC. The Structural Equation Modelling by AMOS 25 was used to test the proposed hypothesis. The results indicate that YHQC acts as a significant factor in enhancing SPE, and UIC is found to act as a partial mediator in this relationship. This study has contributed to the literature and practices by presenting a comprehensive quality culture from Yanpei Huang’s perspective, confirming the above relationship, and providing practical suggestions for stakeholders to develop a quality culture in TVET institutes, promote UIC, and enhance SPE.

## 1 Introduction

The changing trends in the global education market have altered expectations of students seeking more than just skills and knowledge, i.e., ways to deal with other challenges, including labor market dynamics (Tomlinson, 2012) and employability prospects (Pitan and Muller, 2019). Nowadays, employee behaviors are guided by their perceptions rather than the objective elements of employability (Baluku et al., 2021). Employability indicates individual characteristics that foster adaptive cognition, behavior, and affect, enhancing the individual-work interface (Fugate et al., 2004). From a more holistic view, McQuaid and Lindsay (2005) argued that employability should include individual factors, personal circumstances, and external factors, acknowledging the importance of supply- and demand-side factors. Employability is defined as “a set of attributes, skills, and knowledge that all labor market participants should possess to ensure they have the capability of being effective in the workplace to the benefit of themselves, their employer, and the wider economy (CBI, 2009, p. 8).” After its early inception into the mainstream literature in 1909, the concept of employability has commanded a central place in the U.K., European states, and many other nations (McQuaid and Lindsay, 2005; Bakar et al., 2013). Employability becomes even more crucial in highly populated countries like China, where vocational institutes release 10 million highly skilled professionals into the labor market annually (Li et al., 2023). In 2022, there were 7,201 secondary vocational schools with 3,392,900 students, 32 vocational schools at the undergraduate level with 228,700 students, and 1,489 higher vocational schools with 16.7 million students (Ministry of Education of the People's Republic of China, 2023). SPE depends on the context and evolves with society and people over time (Williams et al., 2016; Sánchez-Queija et al., 2023). Researchers have extensively explored the determinants of students’ perceived employability (SPE) in different university settings (e.g., Clarke, 2018; Pitan and Muller, 2020). Even though vocational education is vital for socio-economic (Ekhalia et al., 2021; Olabiyi, 2023) and sustainable development (Paryono, 2017) and TVET institutes are different from traditional universities, less is known about the determining factors of SPE in vocational education (Sánchez-Queija et al., 2023).

Of available explanations, experts argue that quality culture fosters the development of high-quality assurance systems (Hildesheim and Sonntag, 2019; Thai et al., 2022) through permanent quality enhancement across the entire institution (EUA, 2006). Quality culture represents the collective mindset, values, and practices, prioritizing high standards of teaching, management, and overall program quality in vocational education. Graduates from reputable institutions have better employment prospects and are more employable than those graduating from less reputable institutions (Qenani et al., 2014; Chen et al., 2015; Drydakis, 2016; Ergün and Şeşen, 2021). Experts explain that high-ranking institutes nurture unobservable characteristics among students, such as self-efficacy, self-confidence, transferable skills, and commitment that employers need from potential employees (Oluwajodu et al., 2015; Drydakis, 2016). Thus, academic institutions worldwide strive to develop a robust and culturally aligned quality culture that facilitates an understanding of the roles and responsibilities of each stakeholder in institutional quality development (Thai et al., 2022). Since quality culture may vary across countries and institutes, empirical findings based on western quality culture or traditional university instruments are less likely to accurately capture the effects of quality culture on SPE, particularly in Chinese vocational education.

The debates on quality culture within vocational education in China can be traced back to the seminal work of Yanpei Huang (1878–1965), the founder of modern Chinese vocational education, who laid the ideological foundation of vocational education in China (Chen, 2016; Su et al., 2021). In 2004, the Ministry of Education published “Several Opinions on Deepening the Reform of Higher Vocational Education with Employment as the Orientation.” Recently, President Xi stressed the need to “promote the integration of vocational education, industry education, and science education, and optimize the positioning of vocational education types; accelerate the development of national strategic human resources, and strive to train more experts, outstanding engineers, craftsmen, and highly skilled talents (Xi, 2022).” This viewpoint mirrors the key tenets of a robust quality culture in TVET institutes proposed by Yanpei Huang (hereafter YHQC). YHQC offers a comprehensive road map for TVET institutes to cultivate positive employability perception among students by promoting practical problem-solving and masters of hands-on skills and initiating university-industry collaboration (UIC) for superior outcomes (Zhang, 2021). Despite such theological influence, apart from a few concept papers, the effects and potential effects (e.g., SPE or UIC) of YHQC have not been empirically validated in China and beyond.

Importantly, as an intermediary mechanism, UIC promotes interactions (knowledge and technology exchange) between parts of the higher education system and industry (Ankrah and Al-Tabbaa, 2015). With UIC playing a significant role in improving vocational education quality in China (e.g., Artess et al., 2017; Jin, 2023; Li, 2023; Lu, 2023; Shi and Guo, 2024), universities and industries should join hands to enhance skills, abilities, and employability of students (Pinto and Ramalheira, 2017; Brachem and Braun, 2018). Currently, the low engagement of Chinese enterprises in UIC programs (Jin, 2023) reflects poor coupling and coordination. Some vocational institutes have failed to deliver quality graduates that meet enterprise standards (Jacob and Gokbel, 2018). With a few exceptions, previous studies on SPE mainly studied factors such as career development learning (Ho et al., 2023), mentoring (Niu et al., 2024), academic engagement and stress (Ma and Bennett, 2021), experiential learning activities (Pitan and Muller, 2019), academic engagement and stress (Ma and Bennett, 2021), generic skills, academic performance, personal circumstances, and external labor market (Ergün and Şeşen, 2021), the role of teaching staff (Petruzziello et al., 2023), students’ perceived organizational support (Trullas et al., 2018), psychological capital (Ayala Calvo and Manzano, 2021), career ambition, university reputation, university commitment, technostress related to technology-enhanced learning, and mental well-being (Schettino et al., 2022). As discussed above, the intermediary role of UIC, particularly between YHQC and SPE in the vocational education setting in China, remains widely unexplored.

A critical review of the existing literature highlights the following knowledge gaps. Firstly, many scholars have compressively examined SPE in conventional university contexts. With the modern vocational education contexts being widely unattended, particularly in China (Sánchez-Queija et al., 2023), there is an imminent need for exploring the antecedents to SPE across different regions and cultural contexts (Pitan and Muller, 2020). Secondly, although the seminal works, philosophy, and contributions of Yanpei Huang have significantly influenced the theory and practice of TVET in China, there is a lack of empirical studies confirming the potential role of YHQC in shaping SPE (Su et al., 2021; Zhang, 2021), particularly in Chinese setting (Chen, 2016). Thirdly, many academics, educators, and institutions endorse UIC as a critical factor in the quality of vocational education, but only a few studies have empirically validated its mediating role in the quality culture-SPE nexus (Ankrah and Al-Tabbaa, 2015; Artess et al., 2017). Importantly, UIC is vital in equipping students with the technical skills and competencies different industries require (Cai and Kosaka, 2024). Since the majority of studies have adopted instruments of quality culture designed for conventional and Western university settings, they are less likely to precisely capture the distinct features of quality culture in diverse cultural contexts (Hildesheim and Sonntag, 2019; Thai et al., 2022), e.g., Chinese TVET settings. Fourthly, the rapid shift (e.g., technological, social, and environmental) within the educational industry requires adopting an inclusive, interdisciplinary, and integrative approach to explaining various cognitive mechanisms and the interplay of factors. However, only a limited number of scholars have constructed integrative models combining multiple theories and concepts (e.g., Human Capital Theory, Total Quality Management, and the Triple Helix Model) to offer in-depth insight into factors impacting SPE (Becker, 1975; Deming, 1982; Etzkowitz and Leydesdorff, 2000), particularly in diverse and distinct TVET contexts worldwide.

Therefore, the primary purpose of this work is to empirically translate YHQC into a comprehensive quality assessment construct and then measure its direct and indirect effects (through UIC) on SPE among students in a Chinese vocational institute. The present inquiry is theoretically significant in the following ways. Firstly, the paper links the human capital theory (HCT) from labor economics to TVET, thereby inciting fresh debates in the SPE literature. Secondly, the paper bridges the gap between TVET and the total quality management (TQM) literature by providing measurable metrics (YHQC) to assess quality culture among vocational institutes while linking them to SPE and UIC. Huang stresses accessibility, social connection, equity, and employment in TVET, echoing TQM principles of providing quality products to meet stakeholder expectations of students and employers (Tarí et al., 2020) and continuous enhancement of education quality through certain practices, e.g., quality culture and UIC. Thirdly, the study builds on the triple-helix model (THM) (Etzkowitz and Leydesdorff, 2000; Etzkowitz and Zhou, 2017; Cai and Amaral, 2022) to empirically establish the mediating role of the UIC in linking institutions (academia and industry). The models support TVET’s function as the bridge, preparing students for the labor market with industry partners’ participation and government support.

## 2 Literature review and hypotheses development

### 2.1 Theoretical foundations

The current integrative framework is built on three influential paradigms and theories: (i) HCT (Becker, 1975); (ii) total quality management in education (TQM) (Deming, 1982); (iii) THM (Etzkowitz and Leydesdorff, 2000). The HCT provides critical insight into TVET institutes’ role in cultivating quality human capital. Quality vocational institutes provide the economy with the most vital resources (human capital) by equipping students with specialized skills, improving their job prospects, and providing human resources for economic and industrial development. Next, applying TQM practices enables TVET institutes to cultivate high-quality human capital by providing quality education to meet the demands and expectations of different stakeholders, e.g., students, employers, industry, and governments. Therefore, TQM is central to the TVET strategic objectives in ensuring quality continuous improvement through various practices (e.g., maintaining high standards of program quality) to meet the changing needs of individuals (employability), industry, and the economy. For this purpose, TVET institutes must continuously interact and collaborate with related industries to guarantee high employment rates. Thirdly, the THM explains the enabling mechanism through which TVET institutes can proactively engage with industry and government stakeholders to fill gaps between industry requirements and TVET output. With robust UIC initiatives (e.g., improved curriculum and internship), students are more likely to satisfy skills and knowledge criteria set by the industry.

### 2.2 Yanpei Huang and his thoughts on quality culture

On May 6, 1917, Yanpei Huang (also named Yen-Pe’i Huang) and 48 others established the China Vocational Education Association, which extensively investigated the educational, economic, and social conditions at home and abroad and then provided modernized vocational education. On May 15, 1918, Huang and other patriots founded the “most experimental school,” China Vocational School (*Zhonghua Zhiye Xuexiao*). It is the first school with the word “vocational” inside the name in modern China. Huang has produced many publications and documents to explain his thoughts on the quality of vocational education. Therefore, Huang advocated the ideal of “enabling the unemployed to have jobs and enabling the employed to enjoy their jobs” (Tian and Li, 2018, p. 298). Huang advocated that institutes should be concerned about students’ future employment prospects from the beginning of enrollment, considering both students’ individual needs and society’s actual needs (Song, 2008). Besides, Huang mentioned the principles of conducting vocational education: vocational education must be tailored to local conditions and students’ aptitude and oriented towards industry and society (Tian and Li, 2018). The only life of vocational education institutes is socialization (Huang, 2022a). Huang believed that the education sector must communicate with the professional sectors. The programs (majors) are to be established depending on the needs of the professional sectors; the selection of teaching materials needs to consult the professionals for their opinions.

Elaborating on Yanpei Huang’s philosophy, Tang (2007) asserts that the characteristics of the professional world should be integrated into students’ training, given that society and education reside in a connected system. If decoupled from society, education may not yield desirable and practical outcomes. Therefore, the author recommends that those who run vocational schools must communicate and simultaneously liaise with all educational and professional circles. Huang criticized that kind of vocational education, which only focused on cultivating skills but paid no attention to nurturing the spirit, thus turning a good education into an education that only makes students into instruments. Such an education system may create promising apprentices, but they are less likely to cultivate citizens with good cognitive ability, manners, and passion to serve society (Dong, 2007). All students at the China Vocational School were required to write an oath when they enrolled to develop respect for labor and show professional ethics: respect for labor (besides half-day work, all students perform all the cleaning, entertaining, and other work on campus on a rotating basis); abide by the rules (students form the autonomous association, set up all the rules and abide by them on campus); serve the community (students are engaged in services off-campus as well as on-campus services) (Tian and Li, 2018). Li (2024) argues that Yanpei Huang has constructed a vocational education quality culture aimed at achieving “Making a living for oneself and serving the country” by emphasizing the socialization of program designing, scientific management, teaching that integrates work and learning, and cultivating moral literacy.

### 2.3 Quality culture and students’ perceived employability

Graduates in TVET institutes will have better employment prospects if employers recognize their institutes with a good reputation for education quality (Bhorat et al., 2012). A report published by the Wilson (2009) showed a positive correlation between the graduation rate of four-year undergraduate universities in the United States and their competitiveness (Wu et al., 2016). Therefore, American companies can determine the quality of graduates by the university’s graduation rate. Unlike American universities, the graduation rates of Chinese universities are all very high, above 90%. Chinese companies can only judge graduates’ quality by the university’s reputation. A university’s reputation relies on stakeholders’ perceptions of students’ quality, social serviceability, research output, influence, and trustworthiness. Due to their excellent quality programs and interaction with alumina and industry (Drydakis, 2016), reputable TVET universities can develop stronger links with employers to provide internships, job fairs, or similar activities to co-develop students’ professional experience and knowledge. Despite mixed findings in the literature (Finch et al., 2013; Clarke, 2018), evidence suggests that a university and its reputation affect students’ employment and perceived employability (Rothwell et al., 2008; Okay-Somerville and Scholarios, 2017). It can be inferred that TVET universities with good reputations and quality cultures usually produce graduates who are more employable and preferred by employers. The more the students perceive their universities to be good in education quality, the more they become confident about their knowledge, attributes, skills, and abilities (Qenani et al., 2014). Drydakis (2016) finding is similar: graduates who studied in higher-ranked universities, which are guaranteed by institutional quality culture, receive more invitations to interviews and higher entry-level annual salaries. Huang believed the most considerable difficulty in running vocational schools is the way out for students after graduation (employment). No matter how well a school does, recruiting students later will be challenging if the first batch of graduates has no way out (Tian and Li, 2018). More specifically, Huang stated that vocational education aims to develop one’s personality, prepare one to earn a living, serve society, and prepare the country and the world to enhance productivity (Huang, 2022b). It can be inferred that a TVET institute with this quality culture will be dedicated to improving students’ employability. Thus, the first hypothesis of this research is:

Hypothesis 1: Yanpei Huang’s quality culture positively affects students’ perceived employability.

### 2.4 Yanpei Huang quality culture and university-industry collaboration

The notion of quality culture extends beyond the technocratic perspective of quality assurance, focusing on an organizational-psychological paradigm along with structural-formal quality assurance methods. Quality culture emphasizes a shift from traditional perspectives in education (i.e., accountability, regulation, and quality control) to institutional freedom, quality enhancement, and reliability (Bendermacher et al., 2017). It involves a shared commitment to continuous improvement, stakeholder engagement, industry relevance, learner-centred approaches, and the integration of effective teaching methods, assessment strategies, and support systems. TVET universities with a quality culture usually provide good education and technology services, emphasizing and promoting more collaboration with the industry. Their graduates are believed to have acquired the needed knowledge and skills because of the university’s quality culture. Thus, the industry could gain good quality employees after the co-educating programs, and they could have more new ideas from new talents. The faculty in universities with good reputations perform better in collaborative projects, which the industry needs for new products or technology upgrading. Besides, there has been a substantial increase in the literature on UIC (Ankrah and AL-Tabbaa, 2015), leading to pressures on industry and universities (Giuliani and Arza, 2009). Enterprises face challenges from technological development, intense global competition, and shorter product life cycles. Concerning TVET universities, pressures have included the need for practical professional knowledge and skills, funding, and the social responsibility to serve economic and societal growth (Philbin, 2008). Vocational education institutes need to provide high-quality education to students, supporting the development of a skill-based society. These pressures on both parties have led to an increasing impetus for developing UIC to enhance innovation and economic competitiveness at institutional levels through knowledge exchange between academic and commercial domains (Perkmann et al., 2013). From the above discussion, the following hypothesis is proposed:

Hypothesis 2: Yanpei Huang’s quality culture will positively influence university-industry collaboration.

### 2.5 University-industry collaboration and students’ perceived employability

UIC and integrating industry and education are the most distinctive features of vocational education (Liu and Li, 2023), often resulting in knowledge creation and technology transfer, which are beneficial for the industry, academia, society, and government (Abbas et al., 2021). The university-industry collaboration has gained widespread interest because of the high degree of innovation and economic growth (Ivascu et al., 2016), and there is an excellent urge for robust partnerships between them. The collaboration between industry and academia promotes the interaction between the educational system and professional sectors, aiming to enhance the program quality. It helps professional sectors develop new products and services, train and develop employees, and recruit new talent, and TVET institutes (graduates) output acts as industry input. Considering the close relationship, the industry should participate in and collaborate with TVET institutes to achieve common objectives, like cultivating talents. UIC can benefit students through enhanced employability, awareness of industry trends, and research grants (Lutchen, 2018). UIC will promote industrial internships and other activities, thus performing skill-based learning, blended learning, and experiential learning, which lead to workplace exposure. Internship and experiential learning activities include students’ exposure to work experience and other real-world activities (industry visits, community service, and career exhibitions), enabling students to make connections between theoretical academic knowledge and practice in the workplace (Pitan and Atiku, 2017). Such initiatives enhance employability. Consistent with Ishengoma and Vaaland's (2016) statement, UIC includes training and education (e.g., cooperative education and industrial training), services and consulting (e.g., modernizing programs and sabbatical), research, activity-based sponsoring (e.g., shared equipment or facilities, equipment donations). Industrial involvement in designing curricula and industry-driven student–industry projects are the key drivers for students’ practical skills development, which can significantly enhance students’ employability attributes. Many employability studies (Qenani et al., 2014; Jackson and Wilton, 2017; Oliver and Jorre, 2018) established that students’ participation in experiential learning activities gives them a competitive edge in terms of obtaining employment after graduation. For example, Qenani et al. (2014) revealed that SPE increased by 250% through participation in experiential learning activities, particularly internships in America. Anand et al. (2016) showed that South African graduates without work experience are less likely to find graduate-level positions. Assuming that students are aware of the positive link between UIC and employability, the greater their exposure to industry, the higher their level of SPE and vice versa. Based on the above debate, it is proposed that:

Hypothesis 3: University-industry collaboration has a positive influence on students’ perceived employability.

### 2.6 The mediating role of university-industry collaboration

Evidence suggests that a few mediating factors can explain the effect of YHQC on SPE. The theory exists that UIC mediates the relationship between the quality management system and SPE (Abbas et al., 2021). Through the UIC, students get more opportunities to be exposed to the real professional world and gain more attributes needed by work. Thus, students have better expectations of their employability. In our common sense, a university with a quality management system comes with its quality culture. Therefore, it can be inferred that the UIC may mediate the relationship between quality culture and SPE. From the previous discussion on the influence of YHQC on UIC, it is apparent that students from universities with quality cultures can build their portfolios on experiences from the real world, enhancing their employment opportunities (Drydakis, 2016). Consequently, the current study investigates whether UIC mediates the relationship between YHQC and SPE and whether the effect of YHQC on SPE is meaningfully improved with the introduction of the mediator. Therefore, the fourth hypothesis is:

Hypothesis 4: University-industry collaboration mediates the influence of Yanpei Huang’s quality culture on students’ perceived employability.

**Figure 1:**
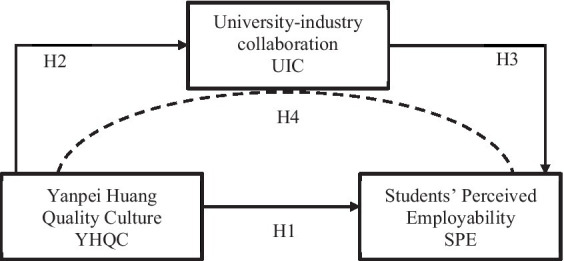
Proposed theoretical framework.

The relationships among the three variables proposed in the hypotheses above are illustrated in the theoretical framework shown in Figure 1.

## 3 Methodology

### 3.1 Participants and procedures

Three hundred sixty-six students from one TVET university in China participated in the study. Twenty-five questionnaires were incomplete. Three hundred and forty-one questionnaires were analyzed. From a contextual perspective, the selection of the university was based on convenient sampling and other criteria discussed hereafter. Originating in the 1950s, the university offers a three-year education to more than 9,000 students, upholding the belief of “born for the city, living for the city.” The university is dedicated to “putting education as a foundation, promoting cross-border integration, servicing the demand, and pursuing excellence.” It aims to cultivate high-level artisans and skilful talents who love craftsmanship and professional ethics. The students are mainly from the province where the university is located, as with other vocational universities. The university provides majors in finance, management, computer science, transportation, construction, tourism, education, designing. This vocational institute is located in an urban centre. After the Ministry of Education recognized it as “the Good Quality Vocational College” in 2019, it was certified as “the Exemplary Modern Higher Vocational College by the Department of Education” in 2020. Out of 50 TVET institutes in Fujian, the university has retained its rank among the top 6 TVET universities in the region. Follow the requirements of “Law on Vocational Education of the People’s Republic of China” (2022), “Notice on Issuing the Work Plan for Vocational Education Reform in Fujian Province by Seven Departments including the Education Department of Fujian Province” (2019) and “Notice of the General Office of the People’s Government of Fujian Province on Several Measures to Deepen the Integration of Industry and Education and Promote the High-quality Development of Vocational Education,” this university have carried out many practices to accurately align with industry demands and cultivate high-quality technical and skilled talents. The above credentials, background, and passion for quality education make this institute an ideal candidate for a case study.

For data collection, information on participants’ characteristics, perceptions of their university’s quality culture, engagement in UIC, and perceived employability were collected through a questionnaire. The online questionnaires were distributed to targeted classes by the researcher team in January 2024. Although senior students’ participation was encouraged because of their institutional experience, knowledge, and familiarity with the university culture, the completed survey document was representative of 89 first-year students, 107 s-year students, and 145 third-year students. Participants responded to the questionnaire after being informed of the research aims and voluntary participation. The summary characteristics of the respondents are shown in Table 1.

**Table 1:** Participants’ demographic information.

Demographic	Gender	Number
Gender	Male Female	201 140
Grade	First-year Second year Third year	89 107 145
Discipline	Engineering and Construction Finance and Management Education	158 120 63

### 3.2 Measures

The construct details are as follows. The YHQC, the independent variable, was measured with a 5-point Likert scale containing 21 items, adopted and revised from Huang’s writings (Huang, 2022a,b). The scale included five constructs: leadership, strategy, resources, people, and process. Students were asked to rate their perception of the university’s quality culture by selecting 1 to 5: “1” indicated strong disagreement; “5” indicated strong agreement. The example items constitute “My university’s leadership ensures vocational education is accessible and promotes inclusivity, reflecting our commitment to social responsibility,” “My university’s strategy emphasizes the practical application of knowledge and skills, aiming to solve real-world problems,” and “Our teachers possess enough practical experience to prepare students for professional environments.”

UIC, the mediator, comprised an empirically validated 5-point Likert scale, including 17 items adopted from the works of Ankrah and AL-Tabbaa (2015). These items can be categorized into five constructs: meeting and network, communication, training, personal mobility, and employment. Students were asked to rate their perception of the engagement in UIC by selecting a number on the scale. Some example items are “I have participated in academic and professional gatherings that align with my vocational discipline, including conferences and workshops,” “There are (co)publications of research papers, reports, and booklets,” “Communication with industry professionals through various channels (voice, email, conference calls) is a regular part of my academic experience,” “Customized educational programs developed by industry partners have been a significant aspect of my training” and “The exchange program between our institute and industry facilities for staff and students has benefited my education.”

SPE, the dependent variable, containing 16 items, was taken from Rothwell et al. (2008). Students were asked to rate their perception of their perceived employability in terms of the skills, knowledge, and abilities they have acquired through their university studies. They responded to the items on this scale by selecting a number on a 5-point Likert scale – with “1” indicating strong disagreement and “5” indicating strong agreement. The example items are “I achieve high grades in my studies,” “I regard my academic work as a top priority,” and “Employers are eager to employ graduates from my university.”

### 3.3 Ethical clearance

The authors applied for ethical clearance from the sample university. The application was granted full ethical approval. All student participation was voluntary, and anonymity and confidentiality of the collected information were assured.

## 4 Results and findings

The study empirically examined the effect of YHQC on SPE while simultaneously estimating the mediating role of UIC in the same equation. EFA was tested. Then, the path model of the study was assessed using AMOS 25. The measurement model was evaluated, and then the structural model was examined.

### 4.1 Exploratory factor analysis

**Figure 2:**
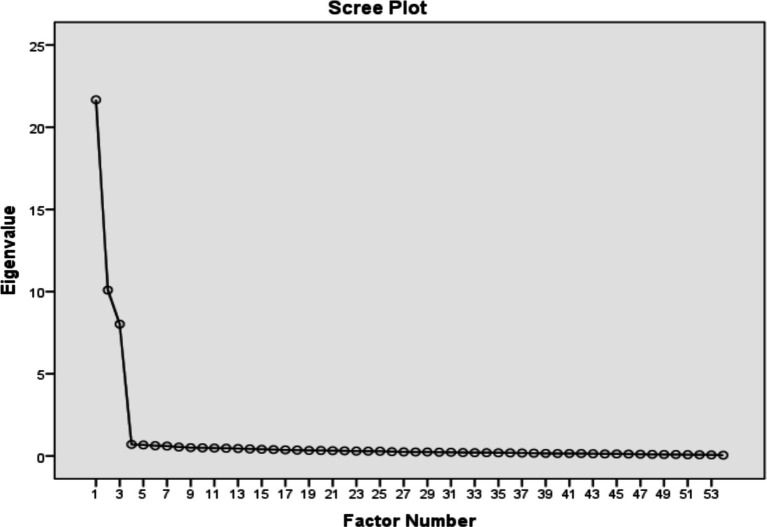
Scree plot.

Prior to the confirmatory factor analysis, the exploratory factor analysis (EFA) on 54 items was conducted to explore the number of underlying factors among the selected items. Three factors were tentatively extracted using an EFA with principal axis factoring and varimax rotation. The Kaiser Meyer-Olkin (KMO) measure of sampling adequacy was 0.962, indicating a sample sufficient for factor analyses. Bartlett’s test of sphericity (chi-square = 21767.420; *p* < 0.001; df = 4,131) suggested patterned relationships among the items. Several approaches have been proposed in the literature to determine the appropriate number of factors to extract from the dataset. The most common being eigenvalue and scree plot. The principal axis factoring with varimax rotation retained three significant factors of 54 items, representing the study’s variables. Table 2 shows the 3-factor solution for 54 items derived from the principal axis factoring with varimax rotation, indicating a total variance of 72.132%. Examining the scree plot (*cf.* Figure 2) and Eigenvalues indicate a three-factor solution (*cf.* Table 2).

**Table 2:** Eigenvalues and EFA outputs.

**Total variance explained**									
Factor	Initial eigenvalues			Extraction sums of squared loadings			Rotation sums of squared loadings		
	Total	% of Variance	Cumulative %	Total	% of Variance	Cumulative %	Total	% of Variance	Cumulative %
1	21.666	40.122	40.122	21.386	39.604	39.604	14.785	27.380	27.380
2	10.089	18.683	58.805	9.823	18.190	57.794	12.613	23.357	50.737
3	8.023	14.857	73.662	7.742	14.338	72.132	11.553	21.395	72.132

Extraction method: principal axis factoring.

### 4.2 Measurement model

**Figure 3:**
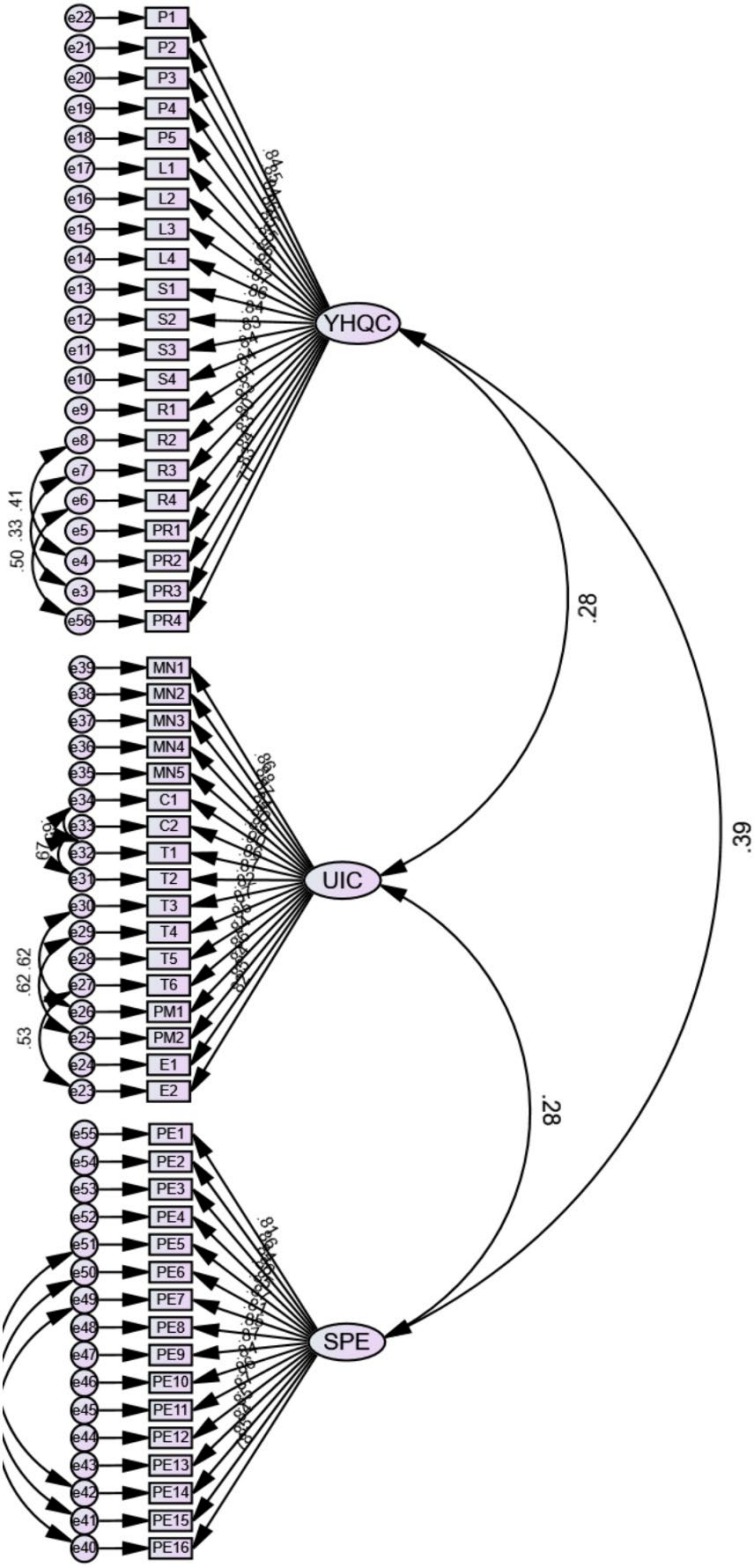
CFA factors load value.

The measurement model shows the relationship between latent variables and their observable items. The confirmatory factor analysis (CFA) was used to estimate the model’s reliability and validity. The estimates obtained through CFA were Cronbach Alpha, factor loading, and average variance extracted (AVE) (*cf.* Figure 3). In the present study, the measurement model comprised 21 items for YHQC, 17 observable items for UIC, and 16 items for SPE.

Per Hair et al. (2011) and Hair et al. (2019), for a model to be reliable, the values of Cronbach alpha shall be greater than 0.70. The results obtained through AMOS 25 revealed that all construct Cronbach Alpha values were above the minimum threshold of 0.70 (*cf.* Figure 3), suggesting that all constructs in the study were reliable. The values of factor loading and AVE were extracted to estimate convergent validity. Consistent with Hair et al. (2022), for an item to be reliable, the values of the corresponding factor loading shall be greater than the minimum threshold of 0.70, whereas AVE for each construct shall be above 0.50. The results obtained in this study revealed that all items’ factor loading and the AVE of each construct (*cf.* Table 3 below) were above the minimum thresholds stated above, reflecting acceptable convergent validity. In addition to the above, discriminant validity was estimated through Fornell and Larcker's (1981) criteria, which suggests that the constructs’ variance with corresponding indicators shall be higher than its variance with other constructs. The study results revealed that the square root of the AVE of each construct was greater than the constructs’ correlation with other constructs of the model.

**Table 3:** CFA validity and reliability.

Constructs	C.R.	AVE	MSV	MaxR(H)	YHQC	UIC	SPE
YHQC	0.980	0.704	0.151	0.981	0.839		
UIC	0.979	0.732	0.080	0.980	0.278***	0.856	
SPE	0.975	0.713	0.151	0.976	0.389***	0.284***	0.844

Discriminant Validity: the AVE for Leadership is less than the MSV. The AVE for Resources is less than the MSV. ***p < 0.001. AVE, average variance extracted; C.R., composite reliability; MaxR(H), maximum reliability (H); MSV, maximum shared variance; SPE, student perceived employability; UIC, University-Industry Collaboration; YHQC, Yanpei Huang’s Quality Culture.

After assessing the measurement properties, the model fit indices were examined. The measure of fit indices is normative fit index (NFI), goodness-of-fit index (GFI), comparative fit index, chi-square/degree of freedom (CMIN/DF), root mean square error of approximation (RMSEA), and standardized root mean square residual (SRMR). According to Bagozzi et al. (1998), for a model to fit, the chi-square/degree of freedom (CMIN/DF) values shall be less than 3. The results revealed that the (CMIN/DF) value was 1.592 (*cf.* Table 3), less than the acceptable threshold. The standardized root mean square residual (SRMR) value was equal to 0.0353, a statistically acceptable range. The results of the other fit indices, such as CFI = 0.963, NFI = 0.906, GFI = 0.810, and TLI = 0.961 (*cf.* Table 3), revealed that the model fitted the data, where all values were in the acceptable range (Bagozzi et al., 1998; Hu and Bentler, 1999; Bryne, 2010). Also, the values of RMSEA = 0.042 (*cf.* Table 3) were in an acceptable range, supporting that the model fitted with the data well (Cudeck and Browne, 1992). In sum, the results of GFI, NGI, RMSEA, SRMR, and CFI in Table 3 complied with the minimum threshold value requirements recommended by several statisticians (Cudeck and Browne, 1992; Bagozzi et al., 1998; Hu and Bentler, 1999; Bryne, 2010).

### 4.3 Hypotheses evaluation

After assessing the measurement properties and goodness of fit of the measurement model, the study’s hypotheses were examined through SEM using AMOS software. The study results revealed that YHQC’s relationship with SPE was positive and significant, thus supporting H1. As predicted in the H1, YHQC enhanced favorable SPE among the sampled students (β = 0.34; S.E. = 0.044; composite reliability (C.R.) = 6.213; *p* ≤ 0.05) (*cf.* Table 4). This result supported prior beliefs that quality management practices contribute to superior student-related outcomes, e.g., SPE (Papanthymou and Darra, 2017), learning (Martin, 2018), knowledge, skills, abilities, and personalities (Abbas et al., 2021). The above outcome is contrary to a Turkish study, which found that the university’s contribution has no positive effects on the perception of employability (Ergün and Şeşen, 2021). That said, SPE is determined by personal (e.g., academic performance, skills and abilities to get a job) and contextual factors (e.g., labor market conditions, the strength of a university’s brand, and demand of the subject area) (Rothwell et al., 2008; Vanhercke et al., 2014). Context is essential to forming perceptions of one’s ability to obtain and maintain a job (Donald et al., 2018; Baluku et al., 2021). Students have better employability in institutes that make more efforts to train students with needed attributes, like thinking critically and creatively, communicating effectively (Brewer, 2013; Desai et al., 2016), coordination skills (Hernandez-de-Menendez et al., 2020), learning and adapting, solving problems independently, using basic technology, and leading effectively (Brewer, 2013).

**Table 4:** Goodness of fit of measurement and structural model.

Indices	GFI	NFI	CFI	TLI	RMSEA	CMIN/DF
Recommended values	≥0.8^2^	≥0.9^2^	≥0.9^2^	≥0.9^2^	≥0.8^3^	
Measurement model	0.810	0.906	0.963	0.961	0.042	1.592
Structural model	0.963	0.906	0.963	0.961	0.042	1.592

CFI, comparative fit index; CMIN/DF, chi-square minimum discrepancy divided by degrees of freedom; GFI, goodness of fit index; NFI, normed fit index; RMSEA, root mean square error of approximation; TLI, Tucker-Lewis index.

Furthermore, the data analysis revealed that YHQC’s relationship with UIC was positive and significant (*β* = 0.28; S.E. = 0.049; C.R. = 5.119; *p* ≤ 0.05); thus, H2 was supported. This output echoed Rybnicek and Königsgruber (2019) finding that institutional factors like resources, structure, willingness to change, and process are critical for successful collaboration between universities and industry. Also, the above results supported the idea that human resources (including leaders and people) play a vital role in the successful implementation of university-industry projects (*cf.* Myoken, 2013; Albats et al., 2020; Ćudić et al., 2022).

For intermediary factors, the data reflected that the link between UIC and SPE was positive and significant (*β* = 0.171; S.E. = 0.048; C.R. = 3.578; *p* ≤ 0.05) (*cf.* Table 4). Thus, the H3 of the study was supported. This finding confirmed Ankrah and Al-Tabbaa (2015), Tran (2016), Huang and Chen (2017), Aliu and Aigbavboa (2021), Borah et al. (2021), and Otache (2022), finding that effective UIC improves graduates’ employability competencies. Vocational education should closely connect with industry, and socialization is essential (Huang, 2022a). With the substantial technological, social, and economic changes in the modern era, the concepts and operations of industrial organizations and TVET institutes have significantly changed. One of the consistent challenges for TVET institutes is keeping pace with knowledge change and complying with industry requirements (Cacciolatti et al., 2017). The positive implications of UIC for SPE lie at the heart of the notion that the industry offers students multiple benefits: (i) extensive mentoring opportunities and exposure to relevant training; (ii) improved job market prospects and professional relevance (Aliu and Aigbavboa, 2021); (iii) enhanced access to a wide range of expertise in product development/commercialization and market knowledge (Sherwood et al., 2004); (iv) high employment opportunities for graduates (Lee and Win, 2004); (v) better professional qualities and contacts needed in the professional work; (vi) provision of experts (faculty) with needed industrial information to teaching which equip and benefit students in the long run. In addition, with more contact with the market, students adapt more to alternative opportunities (e.g., flexible employment, Forstenlechner et al., 2014), improving their perception of future jobs. Besides, UIC can help institutes design industry-oriented curricula and train students in pace with industry dynamics, which gives students a better industry perspective and career options. The more information about the industry and needed attributes students acquire, the more confident they are about employment.

Besides, the mediating role of UIC was tested using Preacher and Hayes's (2008) method. The results revealed that UIC mediated the relationship between YHQC and SPE; thus, H4 was supported (*β* = 0.19; S.E. = 0.048; C.R. = 3.578; *p* ≤ 0.05) (*cf.* Table 5). As quality management is an essential part of quality culture, to some extent, quality management can be seen as quality culture. This finding is similar to that of Abbas et al. (2021), who found that UIC acts as a partial mediator in the positive relationship between quality management and students’ employability in higher education institutes. As employability is seen as one of the measures of an institute’s performance (Knight and Yorke, 2003), TVET institutes have initiated many practices to pursue quality and suitable employment, for example, improving the adaptability of programs, setting a clear strategy of serving the society, providing resources and infrastructures for learning by experience and doing, and especially, building closely connection with industry. Such a university environment with positive industrial and real work-life exposure can promote students’ attitudes toward teamwork, social skills, field knowledge, communication, information and technology, management, creativity and innovation, problem-solving, and critical thinking (Nugraha et al., 2020), intellectual abilities, and self-confidence (Papanthymou and Darra, 2017), ultimately improving their employability.

**Table 5:** Structural model results.

Path	Coeff.	S.E.	C.R.	*p*-value	Decision
H1: YHQC → SPE	0.34*	0.044	6.213	≤0.05	✓
H2: YHQC → UIC	0.28*	0.049	5.119	≤0.05	✓
H3: UIC → SPE	0.19*	0.048	3.578	≤0.05	✓
H4: YHQC → UIC → SPE	0.053*			≤0.05	✓

*p ≤ 0.05. YHQC, Yanpei Huang’s perspective; UIC,University industry collaboration; SPE, Students’ perceived employability; CR, Critical ratio.

**Figure 4:**
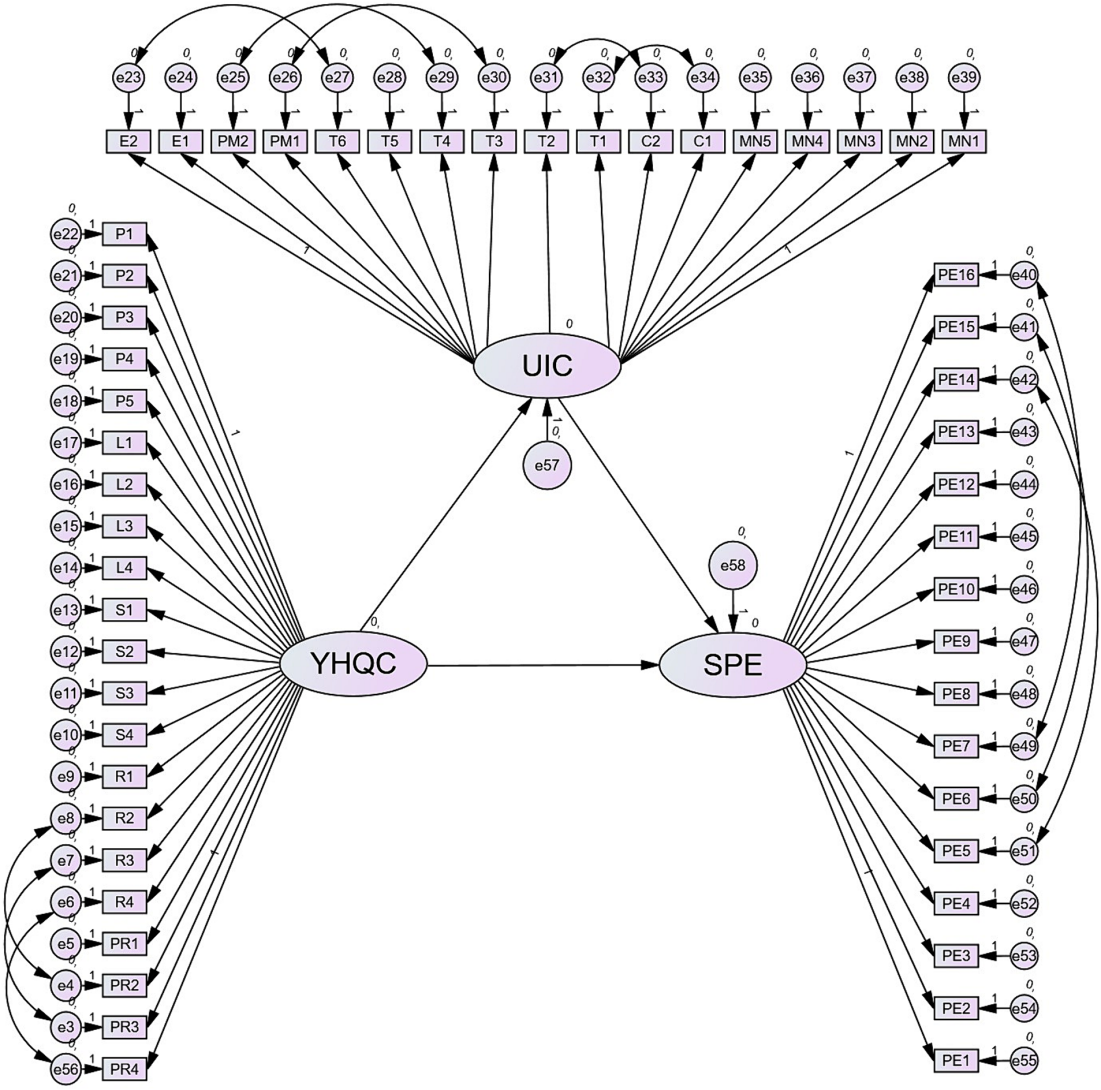
Structural model.

Overall, the results supported that YHQC positively and significantly shaped favorable SPE among the sampled Chinese TVET students, with UIC acting as a partial mediator (*cf.* Figure 4).

## 5 Discussion

Employability is one of the most significant factors that affect students’ choice of where to study (Diamond et al., 2012). By collecting data from one vocational education institute in China, the current investigation empirically validated the positive, significant impact of YHQC on SPE while simultaneously establishing the mediating role of UIC. Facing the intense criticism that graduates could not find proper work due to their inability to develop professional knowledge, expertise, and required characteristics, Huang valued principles like good social interaction, scientific management, faculty with theory and experiences, and learning by doing. Huang preferred to hire faculty with experience, advocating that the programs and textbooks should align with professional practices (Huang, 2019c, 2022a,b). In line with Huang’s philosophy, students valued the importance of hands-on learning, teaching of knowledge and skills required by industry, and learning while doing real work (*cf.* Huang, 2019c). As predicted, the sampled TVET institutes assigned significant importance to the quality of education maintained by the institute and its leadership. They agreed on setting the needed resources and proper procedures to develop practical competencies, skills, and values among students. Echoing Huang’s beliefs, the respondents preferred practical education and competency-based and experiential learning through effective UIC for better employment prospects. As Gentry (1990) stated, experiential learning is mandatory because students may forget what they hear or see but are less likely to forget what they do. As reported by respondents, competency-based education, coupled with UIC experience, could develop and match their knowledge, skills, and abilities with employer demands (Lurie and Garrett, 2017), enabling them to meet the needs of their future job (*cf.* Makulova et al., 2015). As validated by results, students assigned significant value to the importance of dedicated leadership, strategy (socialization), people(teachers), resources, and processes (scientific management) in providing good vocational education. Therefore, it is not surprising that universities with all the above elements in the quality culture prioritize students’ development so their students can have high expectations of their future employment.

### 5.1 Theoretical implications

Theoretically, the current conceptual and empirical framework has several implications. The proposed model connects HCT (labor economics) to TVET, setting a platform for further debates in the SPE literature. Such integration provides a novel viewpoint on how the quality of vocational education improves students’ human capital through skills and knowledge focused on employability. Second, the study bridges the gap between previous literature on TVET and TQM by offering measurable context-specific quality culture metrics, i.e., YHQC. By doing so, the paper examines quality culture in a TVET institution and connects it to UIC and SPE. While affirming the philosophy of Huang for enhancing accessibility, employment, social connection, and equity in TVET education, the data-driven theoretical model aligns with the tenets of TQM, positing continuous emphasis on product quality as per stakeholder expectations and improving the quality of education through various initiatives, e.g., YHQC and UIC (Tarí et al., 2020).

Third, built on the THM (Etzkowitz and Leydesdorff, 2000; Etzkowitz and Zhou, 2017; Cai and Amaral, 2022), the paper establishes the complementary role of UIC in connecting two different sectors, i.e., industry and academia. The empirical framework supports the function of TVET institute as a bridge, equipping students to meet labor market demands with the partnership of industry and government support. Precisely, the THM integration offers an inclusive framework to understand the mechanism through which multi-stakeholder collaborations could improve TVET outcomes. Fourth, the theoretical model enriches existing vocational education literature by identifying and establishing two critical antecedents to SPE in the TVET context of China while simultaneously demonstrating the pertinence of YHQC philosophy and the role of UIC in contemporary TVET settings. Fifth, the paper not only utilizes robust statistical methods (SEM) to establish an integrative theoretical framework based on multiple theoretical concepts (i.e., HCT, TQM, and THM) to explain the YHQC-UIC-SPE nexus, but it also provides empirical support to context-specific (Chinese TVET) research tools (*cf.* Rothwell et al., 2008; Ankrah and Al-Tabbaa, 2015).

### 5.2 Practical implications

The construction of a quality culture needs organizational support and integration of quality commitment and rigid quality management (Xu and Ma, 2017). Quality culture enables institutes to understand the cause-and-effect relationship and to achieve and sustain excellent performance by meeting all stakeholders’ expectations (Abbas et al., 2021). The paper draws from the empirical data to offer several actionable and practical insights for concerned stakeholders, including governments, TEVT institutes, and enterprises.

Governments should introduce more relevant policies to encourage and promote the integration of industry and education. Firstly, government policies and funding should further support the integration of the TVET and industry, aligning with national development plans and goals, like Made in China 2025. For example, the government can further improve the “1 + X” certificate system and give more students professional qualifications during their study period, increasing students’ potential employability. Secondly, the government can establish an information platform to connect TVET schools and enterprises, exchange information, and dock demand between the two sides. Currently, the municipal industry and education consortium and the professional industry and education community have been in practice all over China, and they are expected to channel the academic and industry sides closely. Thirdly, governments can increase investment in TVET and provide more funds and resources to improve teaching facilities, ultimately enhancing student employability and encouraging enterprise active participation in education.

For TVET institutes, embracing a quality culture that focuses on employability is pivotal. Firstly, institutes can construct their quality management system or adopt core principles from Huang’s philosophies, such as emphasizing socialization, respecting labor dignity, and social responsibility in the institute’s ethos. This work encourages policymakers and educational leaders to promote specific initiatives to design, develop, and improve quality culture (i.e., YHQC) in all TVET institutes activities to enhance the readiness of TVET graduates for the Chinese labor markets (Chen et al., 2015; Drydakis, 2016; Government of China, 2022; Ministry of Education of the People's Republic of China, 2023). Integration of quality management systems in higher education institutes boosts the institutional as well as the performance of individual students (Manatos, 2017; Papanthymou and Darra, 2017; Abbas et al., 2021). The TQM can be a valuable tool for pursuing continuous improvement and adaptability. Activities like faculty development programs, industry engagement, feedback surveys, student service and activities, and work-based learning programs can incorporate those values, preparing students for employment and cultivating responsible citizens for society. For example, the industry feedback seminars, industry visits, and questionnaires can facilitate direct communication between the institutes and industry partners, ensuring its curriculum stays aligned with the evolving tech industry needs.

Secondly, in the UIC context, vocational institutes should collaborate closely with local and national industry partners, integrate industry standards, and promote more employability-centric curricula and programs (Pitan and Atiku, 2017; Liu and Li, 2023). Specifically, UIC can include internships in industry, training teachers, joint cultivating students, collaborative research projects, curriculum co-development, and joint construction of training rooms. Thus, students and teachers can learn the knowledge and skills that meet the needs of the industry. This alignment with industry needs is essential for enhancing students’ employability in the rapidly evolving job market. Thirdly, the institutes should develop more programs in new industry sectors, such as green and A.I. technology, emerging service, and green economy, and use the partners’ help in curriculum design to include the latest technological advancements and market demands. In addition to the cultivation of professional skills, institutes should pay attention to the cultivation of communication, teamwork, innovation, and other comprehensive qualities to meet the needs of enterprises. Fourthly, institutes should take the initiative to engage enterprises to participate in universities’ educating activities. Through enterprise visiting, internships, professional competitions, project cooperation, and other forms, students engage in hands-on projects directly. Then, they can learn and apply theoretical knowledge in the actual working environment to improve their career adaptability and employability. Lastly, the findings recommend cultivating and introducing full-time or part-time teachers with rich industry experience and good teaching ability and setting different faculty development programs to encourage teachers to participate in enterprise practice, understand the latest industry trends, and improve teaching quality.

For industry, the results assert the need for enterprises to take the social responsibility of educating skilled talents and actively collaborate with the TVET institutes. Firstly, the industry should actively provide internship and employment opportunities to provide students with a platform for practice and employment. At the same time, they select and cultivate suitable talents for the company’s sake. Secondly, enterprises can provide relevant equipment and resources, participate in the course design and teaching process, introduce practical cases and experiences of the industry into teaching, and improve students’ practical ability and professional skills. Thirdly, enterprises can collaborate and utilize the intelligence of faculty and students in TVET to update or create new products and techniques.

## 6 Conclusion

Vocational education is an integral part of the national education system and human resources development, and it is a crucial way to train diversified talents, inherit technical skills, and promote employment and entrepreneurship. The high-quality development of vocational education is essential for promoting national industrial upgrading and economic restructuring. TVET institutes are expected to train more high-quality technical and skilled personnel, skilled craftsmen, and artisans, and the mission is to improve people’s ability to find employment and entrepreneurship, enhancing their ability to get rich. However, TVET graduates’ poor performance in the industry is a long-lasting issue faced by industrialists. The industry still faces problems, such as ineffective teaching and learning in the real world, and TVET institutes face indifferent enterprise partners (Liu and Li, 2023). This study sheds light on the industry’s role by incorporating UIC in the relationship between YHQC and SPE in vocational education. The finding showed that a significant positive impact of quality culture on SPE and UIC partially mediates their relationship.

Despite its significance, the current research has several limitations. For instance, the findings drawn from the sample data from students and faculty in one public TVET university are less generalizable and limited by geographical, institutional, and demographic biases. Of a few mitigation strategies, the paper describes the sample, context, and characteristics of the sample institution, in addition to robustness and sensitivity checks. Since the perception of students concerning quality culture in the sampled institute and their employability could be biased and not represent students in private institutes, future vocational experts can investigate multi-institutional research across different institutions, regions, and stakeholders, like employers. More so, future researchers could enhance sample diversity and longitudinal design to capture more intricate details about the effect of time, educational background, and demographics. The current sample limitation of using a small sample can be addressed by including a larger sample size and students from other institutes. Finally, factors that impact employability include fields of study and the profession’s demand in the labor market (Rothwell et al., 2008). Future research should investigate the influence of other factors, like gender, field of study, and job demands. Future research can further explore the effects of different collaborations (e.g., internships, joint projects, curriculum development, academic and professional gatherings, co-publications, tailored educational programs, internships, and lectures by industry members at universities and vice versa) to enhance the UIC.

## Data availability statement

The raw data supporting the conclusions of this article will be made available by the authors, without undue reservation.

## Ethics statement

Ethical approval was not required for the studies involving humans because following institutional requirements and local legislations, no approval and ethical review were required for this work on human participants. All participants were provided a written informed consent form/statement. The studies were conducted in accordance with the local legislation and institutional requirements. The participants provided their written informed consent to participate in this study.

## Author contributions

HL: Conceptualization, Data curation, Investigation, Methodology, Project administration, Resources, Software, Supervision, Validation, Visualization, Writing – original draft, Writing – review & editing. SK: Conceptualization, Formal analysis, Investigation, Methodology, Project administration, Resources, Software, Supervision, Validation, Visualization, Writing – original draft, Writing – review & editing. MS: Formal analysis, Methodology, Resources, Software, Validation, Visualization, Writing – original draft, Writing – review & editing.
